# Genomic Diversity of Tomato Brown Rugose Fruit Virus in Canadian Greenhouse Production Systems

**DOI:** 10.3390/v17050696

**Published:** 2025-05-12

**Authors:** Gregory C. Fougere, Dong Xu, Jonathan R. Gaiero, Cara McCreary, Geneviève Marchand, Charles Despres, Aiming Wang, Mamadou Lamine Fall, Jonathan S. Griffiths

**Affiliations:** 1 London Research and Development Centre, Agriculture and Agri-Food Canada, 4902 Victoria Ave N, Vineland Station, ON L0R 2E0, Canadajonathan.gaiero@agr.gc.ca (J.R.G.); 2 Department of Biological Sciences, Brock University, St. Catharines, ON L2S 3A1, Canada; 3 St-Jean-sur-Richelieu Research and Development Centre, Agriculture and Agri-Food Canada, 430 Gouin Boulevard, Saint-Jean-sur-Richelieu, QC J3B 3E6, Canada; 4 Ontario Ministry of Agriculture, Food and Agribusiness, 2585 County Road 20, Harrow, ON N0R 1G0, Canada; cara.mccreary@ontario.ca; 5 Harrow Research and Development Centre, Agriculture and Agri-Food Canada, 2585 County Road 20, Harrow, ON N0R 1G0, Canada; genevieve.marchand@agr.gc.ca; 6 London Research and Development Centre, Agriculture and Agri-Food Canada, 1391 Sandford Street, London, ON N5V 4T3, Canada; aiming.wang@agr.gc.ca

**Keywords:** virus, resistance escape, genomic diversity, biosecurity

## Abstract

Tomato brown rugose fruit virus (ToBRFV) is a recently emerged viral pathogen in the *Tobamovirus* genus first observed in 2014 in the Middle East that has since spread worldwide, causing significant losses in greenhouse tomato production. ToBRFV is easily mechanically transmitted and can escape the durable *Tm-2*^2^ resistance gene, facilitating its global spread. Seed companies have identified novel sources of resistance and introduced these resistance traits into commercial cultivars. The identity, number, and mechanisms of these putative novel resistance genes are largely unknown but could be exerting selective pressures on ToBRFV. Here, we report 15 new ToBRFV genomic sequences from Canadian greenhouse production systems in susceptible and novel resistant or tolerant cultivars collected since 2023. We combined these sequences with five other Canadian ToBRFV genomes previously deposited in Genbank and a further five consensus sequences derived from metagenomic-based wastewater monitoring sequence data and conducted phylogenetic analysis. Most Canadian sequences grouped together when compared with 332 publicly available international sequences, but several isolates appeared distantly related, suggesting multiple introductions to Canadian production systems. High sequence identity between samples suggest movement of ToBRFV between independent greenhouses, highlighting areas where biosecurity can be improved. Several novel non-synonymous polymorphisms identified in the p126 and movement protein (MP) open reading frames (ORFs) were unique to Canadian sequences and associated with infection of novel resistant tomato cultivars. Many polymorphisms in the p126 ORF are located in a region of the protein associated with *Tm-1* resistance-breaking isolates of tomato mosaic virus and ToBRFV, but have not been previously reported. Four novel polymorphisms in MP were also identified and do not appear to be associated with sites previously identified as interacting with *Tm-2*^2^ and could be related to other unknown resistance genes. Together, these results confirm the difficulties in preventing the transmission of ToBRFV, identify putative adaptations to novel and existing resistance genes, and emphasize the urgent need for the cloning and characterization of these new sources of resistance to ToBRFV.

## 1. Introduction

Tomato brown rugose fruit virus (ToBRFV; *Tobamovirus fructirugosum*) has caused significant disruption to global greenhouse tomato (*Solanum lycopersicum*) production since its emergence in 2014 [1,2,3,4]. The devastating impact of ToBRFV and its rapid spread worldwide are primarily due to its ability to evade the long-standing resistance mediated by the *Tm-2*^2^ gene [3,5,6,7,8]. The industry and specifically seed companies have rapidly responded to this devastating pathogen through the development of new resistant cultivars, with resistance defined here broadly as the reduction of pathogen accumulation [9,10,11,12]. These new resistant cultivars were developed by integrating novel resistance traits identified through screening populations of wild relatives of domesticated tomatoes including *S. habrocaites* and *S. pimpellifolium* [13,14,15,16,17]. While potentially many new sources of resistance have been described, little is known regarding the genetic nature or mechanism of action of these new resistance genes [8,17,18]. In addition, ToBRFV isolates capable of evading these new resistant genes have already been reported, and ToBRFV outbreaks are still ongoing [15,19].

Genomes of viruses in the *Tobamovirus* genus contain four open reading frames (ORFs) which encode four multifunctional proteins responsible for viral RNA replication, encapsidation, and local and systemic movement [4,8,20]. ORF1 encodes the p126 protein, also known as the small replicase, which is responsible for RNA replication [20]. The p126 protein also functions as an RNA silencing suppressor, which can have further effects on virus–host interactions [21,22]. ORF2 encodes the large replicase protein in conjunction with ORF1, which is expressed through a ribosomal frameshift read-through domain [20]. ORF3 encodes a movement protein (MP), while ORF4 encodes the coat protein (CP). Each viral protein plays key roles in viral replication and can be under intense selective pressures to escape host-based resistance mechanisms [15,23,24]. The *Tm-1* resistance gene recognizes the p126 protein and inhibits viral replication, while *Tm-2*^2^ recognizes the MP and initiates a hypersensitive response to prevent viral spread [7,8,25]. While identities and functions of new resistance genes have not been widely reported in peer reviewed publications, they are expected to be widely used in recently commercialized elite tomato cultivars and can exert selective pressure(s) on the ToBRFV genome [15,17]. Identifying polymorphic regions can provide insight into novel resistance gene function and durability and the ongoing ToBRFV pandemic [26].

Greenhouse tomato production is a large industry in Canada with over $869 million in farm gate values in 2023, and approximately 73% of all greenhouse tomatoes produced in Canada were grown in the province of Ontario [27]. ToBRFV has caused substantial losses to the industry in Canada since the first known case in 2019 [3,28,29]. Since its emergence abroad in ~2014, the most pressing research on ToBRFV has focused on the identification of novel sources of resistance, or on mechanisms of resistance escape [7,13,14,15,16,17,19,24]. Multiple descriptions of the resistance-breaking of *Tm-1* [23,24,30], *Tm-2*^2^ [6,7,31], or novel resistance genes [15,32] suggest that ToBRFV will continue to evade resistance mechanisms. Here, we report multiple complete or nearly complete ToBRFV genomic sequences from resistant and susceptible cultivars from Canadian greenhouses in 2023 and 2024, and identify multiple putative resistance-breaking polymorphisms in the p126 and MP regions.

## 2. Materials and Methods

### 2.1. Plant Material, RNA Extraction, and Sequencing

Symptomatic tomato leaf samples were collected from three commercial greenhouses in southwestern Ontario in 2023 and 2024 (Table 1). Samples consisted of three leaves collected from one individual plant and were stored at −80 °C (Table 1). In addition, symptomatic leaf samples from two individual plants was collected from one commercial greenhouse in Québec in 2021 (Table 1). Total RNA was extracted from 400 mg of leaf material using the TRIzol reagent (Thermo-Fisher Scientific, Mississauga, ON, Canada) following the manufacturer’s protocol. Total RNA was extracted for PCR screening and genomic sequencing; 1000 µg of total RNA was used for reverse transcription using the ProtoScript II First Strand cDNA Synthesis Kit [New England Biolabs (NEB), Whitby, ON, Canada] following the manufacturer’s protocol using the random primer mix. Double-stranded RNA was extracted from samples collected in Quebec (Qc) and sequenced according to Fall et al. 2020 [33]. PCR-based detection of ToBRFV was performed using ThermoPol Taq DNA polymerase (Thermo-Fisher Scientific) and ToBRFV-specific primers (Supplemental Table S1) [2]. qPCR quantification of ToBRFV was performed using the Luna universal probe qPCR Master Mix (NEB), using the same primers as the endpoint PCR and a custom probe (Supplemental Table S1). A plasmid encoding ToBRFV’s p126 was used as a standard, with dilutions ranging from 10^0^ to 10^8^. RNA was sequenced following the protocol of Kubaa et al. 2023 [34]. *Tm-1* allele differentiation was performed using cDNA derived from extracted total RNA and primers specific for the *Tm-1* (AB287296.1) or *tm-1* (AB287297.1) allele (Supplemental Table S1). *Tm-1* allele differentiation was further validated using a CAPS marker developed in Zinger et al. 2025 [30].

Sequencing library preparation used Oxford Nanopore Technologies’ (ONT) cDNA-PCR Sequencing Kit V14 and additional reagents, Maxima H minus reverse transcriptase (Thermo-Fisher Scientific), LongAmp Taq 2X Master Mix (NEB), Thermolabile exonuclease (NEB), RNase OUT (Invitrogen, Burlington, ON, Canada), and AMPure XP Reagent (Beckman Coulter, Mississauga, ON, Canada), following ONT’s protocol for cDNA-PCR sequencing—sequence specific (SQK-PCS111) for library preparation. ToBRFV-specific primers used were designed by Kubaa et al. 2023 [34], with the sixth primer altered to match published Canadian sequences; all primers were used at a concentration of 2 µM (Supplemental Table S1). For addition of the rapid adapter and loading of the flow cell, cDNA-PCR Sequencing V14 protocol was followed (ONT). RNA was loaded into the flow cell and sequenced using a MinION Mk1B sequencer (ONT). The MinKnow software was used to monitor the sequencing reaction (ONT). Sequencing run times were between 2 and 72 h depending on the condition of the flow cell, and the minimum read length was set to 200 bp. Sequence read files are available on the sequence read archive under BioProject PRJNA1256347.

### 2.2. Phylogenetics

Sequenced reads were assembled using SeqMan Ngen version 17.6 (DNASTAR, Inc., Madison, WI, USA). Reads were mapped to the most recently reported Canadian ToBRFV isolate (NCBI accession OQ674194.1 accessed 18 September 2024). The variant analysis tool for Nanopore whole genome was selected to run the assembly with default settings and variant detection mode set to haploid (ONT). Further analysis was only conducted on samples with over 96% genomic coverage. Consensus sequences for each sample were submitted to Genbank (Table 2). MegAlign Pro version 17.6 was used for multiple sequence alignments and phylogenetic tree construction (DNASTAR). Multiple sequence alignments were conducted using the MAFFT algorithm. Phylogenetic trees were computed using maximum likelihood: RAxML-NG with a bootstrap of 1000. Phylogenetic trees used the general time reversible model, and the optimal tree was selected based on Bayesian information criterion (BIC), Minimum theoretical information criterion (AIC), and Minimum corrected theoretical information criterion (AICc). Phylogenetic trees used the complete or nearly complete cDNA genomic sequences of ToBRFV from this study along with all ToBRFV genomic sequences available on Genbank as of 3 December 2024 (Supplemental Table S2). Nucleotide sequence alignments were used to measure the evolution of the ToBRFV with DnaSP version 6 [35]. Diversity plots in DnaSP were generated by calculating nucleotide diversity with a sliding window of 50 bp.

### 2.3. Protein Modeling

Three-dimensional structures of ToBRFV p126 and MP were predicted using the Alphafold2 program on the Neurosnap webserver (Neurosnap, Delaware, DE, USA) [36,37,38]. Protein predictions were based on amino acid sequences of G2-TOV4. Predicted 3D models were visualized using Geneious prime version 2025.0.1 (Dotmatics, Boston, MA, USA).

## 3. Results

### 3.1. Evolutionary Relationships of Canadian ToBRFV Isolates

**Figure 1:**
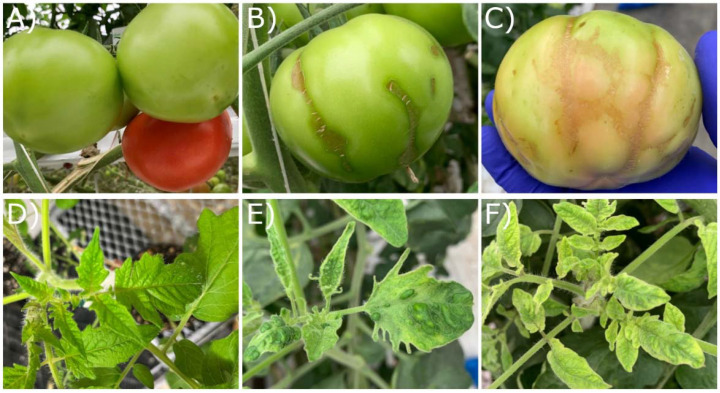
Images of ToBRFV symptoms. (**A**) Uninfected tomato fruit. (**B**) Brown rugose symptoms on fruit. (**C**) Fruit deformations. (**D**) Uninfected leaf. (**E**) Leaf blistering, curling, and deformations. (**F**) Leaf chlorosis and bleaching.

**Figure 2:**
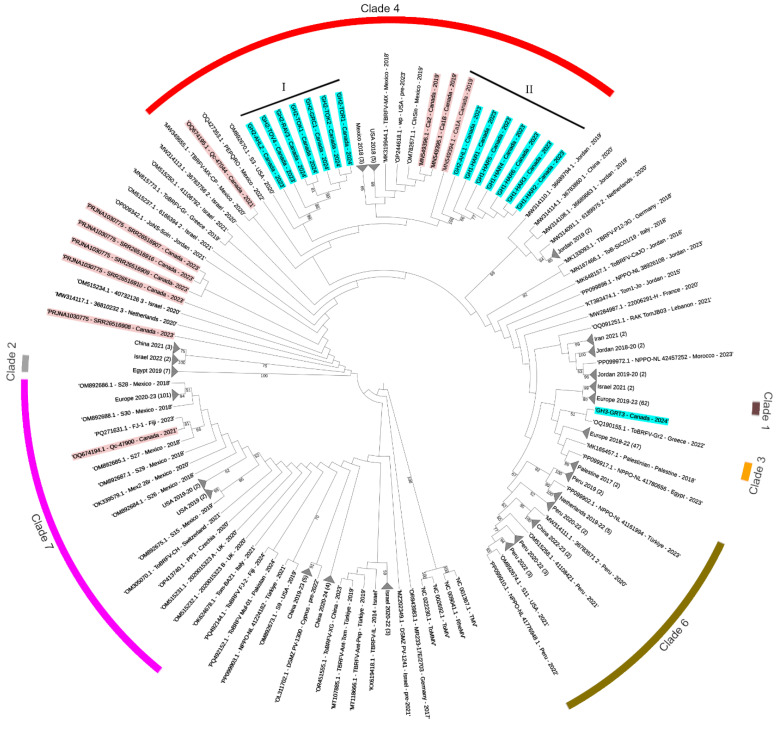
Maximum-likelihood phylogenetic tree of ToBRFV genomes. Sequences highlighted in red indicate Canadian samples collected between 2019 and 2023. Sequences highlighted in blue indicate samples from this study collected in 2023 and 2024. Tobacco mosaic virus, rehmannia mosaic virus, tomato mosaic virus, and tomato mottle mosaic virus were used as an outgroup. Numbers on branches indicate support over 70%. Numbers in brackets indicate the number of isolates collapsed to reduce the complexity of the tree. Clades identified in de Koning et al. 2025 [40], are labeled.

Multiple ToBRFV outbreaks were observed in one commercial greenhouse in Quebec (Qc) in 2021, and three greenhouses in Ontario (On) in 2023 and 2024. Observed leaf symptoms included leaf chlorosis and bleaching, curling, blistering, and growth deformations, while fruit symptoms included brown rugose, incomplete ripening, and deformations (Figure 1). Complete ToBRFV genomes were obtained from 17 different individual symptomatic plants sampled from the 4 greenhouses, consisting of 10 unique varieties (Table 1 and Table 2). Some varieties were labeled as resistant, although the variety names and nature of resistance were kept confidential. Since *Tm-1* has been reported to play a major role in resistance to ToBRFV [10,12,13,24,39], we screened all samples for the presence of the semi-dominant *Tm-1* or recessive *tm-1* allele (Table 1). In total, 7 of the 17 varieties tested were labeled as resistant, and of these, 4 tested positive for at least 1 *Tm-1* allele (Table 1). All samples tested positive for ToBRFV via RT-PCR, except AHL-1 and 2, which tested positive for ToBRFV via qRT-PCR but had lower concentrations of viral RNA detected compared to other samples (Table 1). A further three Canadian sequences submitted to Genbank in 2019 and five consensus sequences assembled from metagenomics-based wastewater monitoring were also included in our global phylogenetic analysis (Table 2; Supplemental Table S2) [28]. These 25 complete or nearly complete genomic sequences were aligned with 332 international complete genomes (Supplemental Table S2). A maximum-likelihood phylogenetic tree was constructed to compare Canadian sequences to the worldwide population of ToBRFV (Figure 2). The most recent Nextstrain analysis of ToBRFV identified seven major clades which were labeled on our tree [40]. The majority of Canadian ToBRFV isolates (n = 18) group together with other North American isolates in Clade 4 (Figure 2). Within Clade 4, sample Qc-47077 collected in 2021 branched closely with a Mexican isolate collected in 2020, while sequences from three Canadian isolates collected in 2019 branched closely with other isolates from Mexico and the United States (Figure 2). New sequences from samples collected in 2023 and 2024 formed two independent groups within Clade 4, largely divided by greenhouse of origin and labeled Group I and II (Figure 2). Group II includes six samples collected on the same date from greenhouse 1, along with one sample from greenhouse 2. Group I contains seven sequences from samples collected successively from June 2023 until November 2024 from greenhouse 2. Outside of Clade 4, the five sequences assembled from metagenomic-based wastewater monitoring largely grouped together, but were not directly associated with other Clade 4 genomes. The sequence from one sample collected in 2021 (Qc-47900) was closely associated with an isolate from Mexico (OM892685.1) and from Fiji (PQ271631.1), within Clade 7 (Figure 2) [40]. A final sequence (GH3-GRT3) branched between Clade 1 and Clade 3, and was more closely related to an isolate from Greece collected in 2022 (Figure 2).

### 3.2. Diversity of Individual ToBRFV ORFs

**Figure 3:**
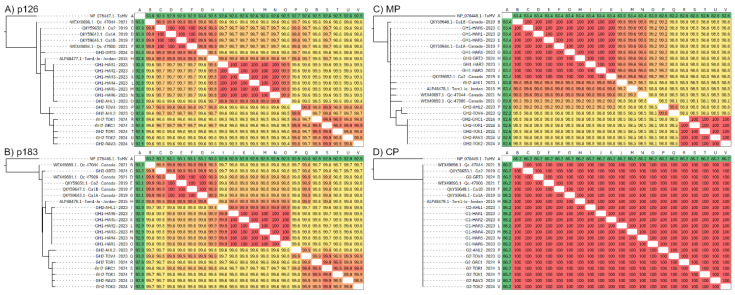
Amino acid sequence maximum-likelihood phylogenetic tree and pairwise analysis of all four ToBRFV ORFs from Canadian samples and the Tom1-Jo reference sequence. (**A**) p126. (**B**) p183. (**C**) MP. (**D**) CP. ToMV was used as an outgroup.

In order to better understand which regions of the ToBRFV genome are undergoing selection in Canadian tomato production systems, the pairwise amino acid sequence identity and phylogenetic relationships of individual ToBRFV ORFs was examined (Figure 3). Three major groups of sequences were observed in p126 phylogenetic trees and shared pairwise identities, which were largely congruent with the whole-genome nucleotide sequence phylogeny (Figure 2). Groups I and II observed in Figure 2 were clearly differentiated in this secondary analysis, and were at least 99.7% identical within group and between 99.5 and 99.6 between groups (Figure 3A). Pairwise phylogenies of the p183 ORF were largely congruent with p126 (Figure 3B). In contrast, MP sequences were not congruent with p126 and p183, separating into two major groups. The second group contained all samples collected in 2024, including GH2-TOR1, GH2-TOK1, GH2-TOK2, GH2-RAV3, and GH2-GRC1, which were all 100% identical (Figure 3C). Notably, samples GH2-TOV4 and GH2-AHL2, which previously branched closely with the five other samples from GH2 in p126 and p183 pairwise analyses, no longer associated with this group in the MP pairwise analysis, with 99.6% identity between the two samples, and 98.5–98.8% identity to the group of five samples (Figure 3C). No major sequence variations were detected within the CP ORF (Figure 3D).

### 3.3. Genomic Diversity of Canadian ToBRFV Isolates

Various measures of nucleotide diversity and selective pressures were analyzed in all 20 Canadian genomic sequences (Table 3). Similarly to previous studies, ToBRFV genomes had low genetic diversity with over 99.3% nucleotide sequence identity between all Canadian sequences (Table 3). A total of 76 polymorphic sites were identified in all Canadian sequences, along with 23 non-synonymous amino acid substitutions (Table 3). The number of nucleotide polymorphisms was highest in the p126 and p183 ORFs with 40 and 51 mutations identified, respectively, while the MP and CP had fewer polymorphisms at 15 and 6, respectively. The number of non-synonymous polymorphisms was also highest in p126 and p183 ORFs with 13 and 14, respectively. The MP had 8 non-synonymous polymorphisms identified, while the CP had no amino acid changes (Table 3). From these Canadian sequences, Fu and Li’s D and F were negative values for the entire ToBRFV genomic region and for all individual ORFs, suggesting population growth (Table 3). The dN/dS ratio was less than 1 (0.16) for the whole genomic region, but was highest for the MP ORF at 0.59, suggesting weaker negative or purifying selection for this region (Table 3).

**Figure 4:**
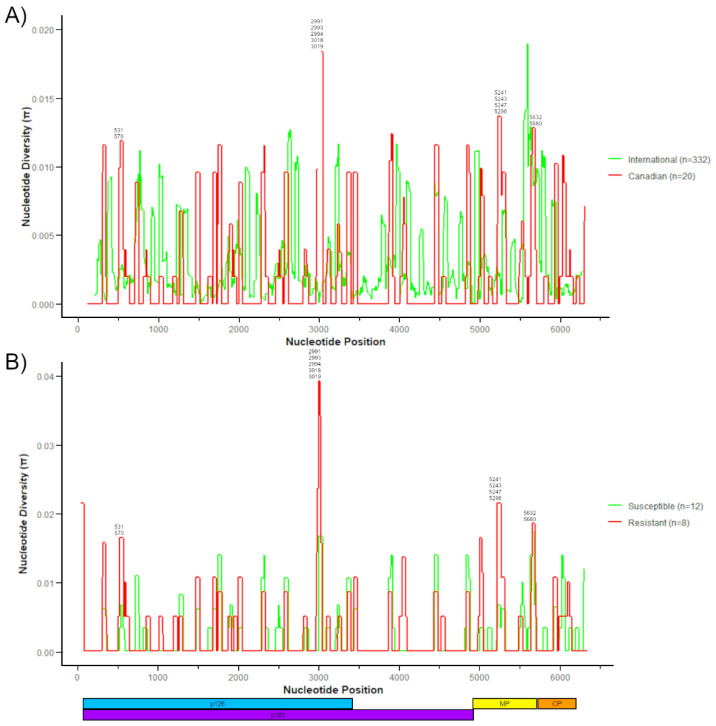
ToBRFV genomic nucleotide sequence diversity (n). (**A**) Graph of nucleotide diversity comparing international sequences (green, n = 337) and Canadian sequences (red, n = 20). (**B**) Graph of nucleotide diversity comparing isolates from susceptible varieties (green, n = 8) and resistant varieties (red, n = 7). A representative diagram of the ToBRFV genome showing all four ORFs is below.

Next, we examined the nucleotide diversity (π) across the whole genome of all 20 Canadian sequences compared to all 332 international genomes to identify polymorphisms unique to Canadian ToBRFV populations (Figure 4A). One major peak was observed in the Canadian samples which was not present in international sequences, including polymorphisms at nucleotide positions 2991, 2993, 2994, 3018, and 3019 that result in non-synonymous amino acid substitutions (Figure 4A, Table 4). Of these five substitutions, two (Y976H and H984Y) have been reported in international sequences while the other three substitutions were unique to Canadian sequences (Table 4). In the MP ORF, one peak from Canadian sequences appeared distinct from international sequences including non-synonymous substitutions at K113N, K114R, R115S, and E132K. Of these four substitutions, three were unique to Canadian sequences, while another mutation at nucleotide position 5296 (E132K) was detected in 7 Canadian sequences and 36 international sequences (Table 4). We also examined the nucleotide diversity of labeled resistant and susceptible cultivars from Canadian greenhouses to identify polymorphisms associated with novel resistance traits (Figure 4B). The major peaks identified in the Canadian vs. international isolate comparison were conserved in the resistant vs. susceptible comparison (Figure 4A,B).

### 3.4. Comparing Canadian ToBRFV Polymorphisms to Resistance-Breaking Polymorphisms

**Figure 5:**
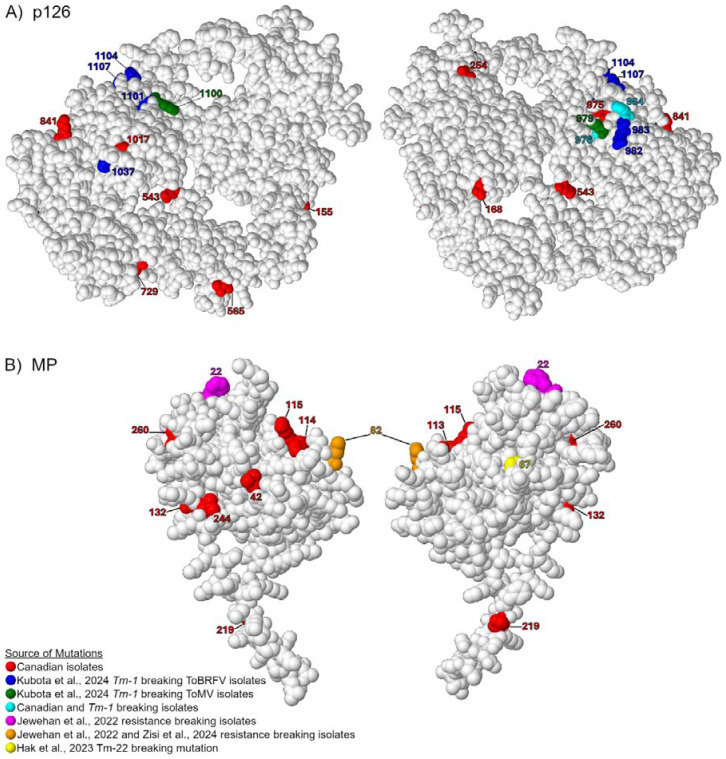
Space-filling molecular model of ToBRFV. (**A**) p126 and (**B**) MP. The legend indicates the color scheme for labeling individual amino acids of resistance-breaking residues and their corresponding citation [7,15,19,24].

Many synonymous and non-synonymous polymorphisms were detected in the p126 and MP regions of Canadian sequences of ToBRFV (Table 4; Supplemental Table S3). Many of these polymorphisms were shared with other international sequences, but some were unique to Canadian sequences. Additionally, many other reports have identified polymorphisms associated with resistance-breaking of *Tm-1*, *Tm-2*^2^, or other resistance genes [7,15,24]. To better understand the variable regions of p126 and MP in Canadian sequences, we mapped the location of these polymorphisms on space-filling protein models of the protein, along with previously reported resistance-breaking polymorphisms (Figure 5) [15,24]. ToMV resistance-breaking isolate Lta1 contained polymorphisms G983G and H984T, while the ToMV1-2 isolate had another two *Tm-1* breaking-associated polymorphisms A1097V and A1100G (Figure 5) [41,42]. A recent report on ToBRFV sequences from symptomatic *Tm-1* plants included four major amino acid changes Y976H, G983E, D1097N, and Y1104F linked to *Tm-1* resistance-breaking [24]. We detected many similar non-synonymous changes in the same region of p126 including H975Y, H975Q, and H984F, which were only associated with Canadian sequences (Table 4). The H984Y mutation was identified in 14 Canadian sequences but was also reported in 32 international sequences (Table 4). For the MP, the C67 amino acid is critical for *Tm-2*^2^ recognition [7]. Other novel resistance-breaking isolates reported one major non-synonymous change at amino acid N82K [15,19]. Here, we identified four non-synonymous changes unique to Canadian sequences (L42S, K113N, K114R, R115S) located proximally to the N82K substitution (Figure 5B; Table 4). Another polymorphism, E132K, was identified in 7 Canadian sequences, but also reported in 36 international sequences (Table 4, Figure 5). Notably, the two polymorphisms at positions 113 and 114 were unique to samples GH2-TOV4 and AHL2, while the L42S and R115S polymorphisms were unique to GH2-RAV3, GRC1, TOK1, TOK2 and TOR1, and these polymorphisms are the primary differentiating factors in the phylogenetic tree and pairwise analysis of the MP (Figure 3C). These sequences were predominantly from samples of cultivars labeled resistant.

## 4. Discussion

ToBRFV has been a major disrupting force since its emergence in 2014 and continues to cause significant losses in Canadian greenhouse tomato production systems. Monitoring the genomic diversity of ToBRFV worldwide can provide important insights into the movement of ToBRFV and help to identify resistance-breaking strains [28,29,40]. Phylogenetic analysis of Canadian sequences suggests multiple introductions into Canadian production systems. We also identified potential transmission between greenhouses and successive adaptations within one greenhouse over a two-year period, potentially due to incomplete decontamination of growing areas or growing susceptible and resistant cultivars together. This further highlights the ongoing risks regarding ToBRFV’s ease of movement into greenhouses and persistence once established. Multiple novel polymorphisms unique to Canadian sequences were identified in the p126 and MP ORFs, suggesting a complex and rapidly changing virus–host interaction environment [43]. The p126 and MP were under purifying selection consistent with ToBRFV under pressure from novel host resistance mechanisms. Amino acid polymorphisms associated with resistance-breaking were identified in the p126 and the MP, suggesting the possibility for new isolates of ToBRFV to evade multiple resistance mechanisms. Together, these results suggest that ToBRFV will continue to be a major issue in tomato production until more durable resistance is identified.

### 4.1. Canadian Sequence Diversity

ToBRFV was first observed in Canada in 2019, and initial samples were sequenced by the Canadian Food Inspection Agency. Here, we sequenced 17 additional samples from Ontario and Quebec greenhouses to better understand the origins and history of ToBRFV in Canadian production systems. Metagenomic-derived consensus sequences [28] largely associated together, but were not closely related to isolates obtained from greenhouses. These wastewater samples were collected from multiple regions in Ontario, at sites ranging over 350 km in distance, and all sampling sites were over 250 km in distance from the Leamington area where most greenhouses are located [28]. The close association of these metagenomic-derived sequences could be due to a short sampling window of approximately two weeks and could be related to overall trends of tomato supply to local grocery stores. Finally, our analysis of ToBRFV genomic sequences from greenhouse production systems only represent three individual greenhouses in Ontario, representing a small proportion of the hundreds of greenhouses producing tomatoes in this province. It is possible that ToBRFV outbreaks from other greenhouses more closely related to the metagenomic-derived sequences were not identified.

Sequences from most Canadian greenhouse samples belong to Clade 4 identified in the most recent NextStrain analysis, while sample Qc-47900 groups within Clade 7, and sample GH3-GRT3 was not associated with a defined NextStrain clade [40], suggesting multiple introductions from international sources. In addition, one isolate from greenhouse 2 branched closely with six samples from greenhouse 1, which could have resulted from transmission between greenhouses. Genomic sequences obtained from greenhouse 2 suggest ToBRFV infectious particles persisted at this site between different plantings and clean outs, showing multiple changes in samples collected successively, which could result in breaking multiple mechanisms of resistance. It is worth noting that at greenhouse 2, susceptible and resistant cultivars were grown simultaneously, potentially facilitating resistance escape. Together, these results suggest that Canadian greenhouse production is under threat from internationally circulating strains and local strains that persist in greenhouses, identifying two areas for improved biosecurity protocols.

### 4.2. Selective Forces on ToBRFV

Since as early as 2021, resistant or tolerant cultivars have been available from seed companies for purchase or through small trial arrangements. More recently, most major tomato seed companies have a wide selection of labeled ToBRFV-resistant cultivars, and they have increased in production acreages as they become available. While many of the polymorphisms identified here were not solely associated with resistant or tolerant cultivars, our results do suggest new selective pressures on the p126 and MP in samples from 2023 and 2024, consistent with more broadly available resistant or tolerant cultivars. Notably, p126 and the MP are the targets of the *Tm-1* and *Tm-2*^2^ resistance genes [25,31]. Four of the seven labeled “resistant” individuals contained a dominant *Tm-1* allele, consistent with this gene playing an important role in the future of ToBRFV resistance [17,24,30]. dN/dS ratios of Canadian sequences indicate negative or purifying selection of these regions, along with limited genetic diversity consistent with previous reports of ToBRFV in North America and most RNA viruses [44,45]. While our results were consistent with previous observations of ToBRFV, we did see an increase in dN/dS values in the p126 and MP ORFs compared to Abrahamian et al., which could a reflect a trend towards positive selection. But these results are difficult to interpret due to the unknown nature of resistance genes and possible presence of multiple resistance genes acting on different viral regions. These results further suggest that p126 could play a key role in viral replication, resistance, and resistance escape, likely related to *Tm-1* which is consistent with other recent reports [12,24,25,30]. *Tm-2*^2^ resistance is not effective at preventing ToBRFV infection, suggesting other gene(s) are involved in the selective pressures on the MP [15]. Characterization and determining the identity of novel resistance genes is critical to developing improved tomato cultivars [12,15,17,46].

The *Tm-1* resistance gene was not widely used in tomato production in recent decades, but this gene has received new interest due to its involvement in ToBRFV tolerance [12,17,24,30]. Recent results have already shown that *Tm-1* is not effective at preventing ToBRFV replication alone [24]. Amino acid substitutions in the p126 region of Canadian sequences have not been previously reported in resistance-breaking isolates of ToBRFV or ToMV and could suggest multiple possible mechanisms of resistance gene evasion [12,23,24]. Novel polymorphisms identified in this study are located in the same region as ToBRFV and ToMV resistance-breaking substitutions identified in Kubota et al. (2024) [24]. The authors suggest a key mutation like the Y976H substitution could reduce the affinity of p126 to *Tm-1*, and further mutations could accumulate to further promote viral replication, which our results also support [24]. We identified four amino acid substitutions in the p126 region and a further four substitutions in the MP region unique to Canadian sequences, suggesting multiple different mutations can overcome *Tm-1*- or other resistance gene-mediated resistance. *Tm-1* is also temperature-sensitive, and the resistance mechanism can break down over 35 °C, further suggesting that *Tm-1* alone is not a reliable source of resistance [13,47].

The MP is another frequent target of host resistance against Tobamoviruses, specifically the durable *Tm-2*^2^ resistance allele [8]. *Tm-2*^2^ encodes a nucleotide-binding leucine-rich repeat (NLR) class immune receptor that recognizes the Tobamovirus MP, with a conserved cysteine residue at C68 required for *Tm-2*^2^ recognition, which ToBRFV evades [7]. The Tobamovirus MP is an important virulence gene and could be transported between cells helping to initiate new infections, representing an important target for host-based resistances [8]. New resistance genes could target the MP. One patent identified a potential ToBRFV resistance gene *SlRGA1*, a putative disease resistance gene on chromosome 8 (Solyc08g075630), and confirmed its role in ToBRFV resistance through a virus-induced gene silencing approach [11]. RGA1 encodes a putative leucine-rich repeat/nucleotide-binding ARC domain-containing protein and has some homology with *Tm-2*^2^, which is consistent with a resistance gene that could have activity against ToBRFV [19,48]. The Tom2M-Jo isolate was reported to evade this novel resistance variety, and two polymorphisms (F22Y, and N82K) could be responsible for this resistance escape [15]. In Canadian sequences, we identified four previously unreported non-synonymous polymorphisms (L42S, K113N, K114R, R115S) which could also be linked to resistance escape, potentially related to this gene. Another MP polymorphism E132K was prominent in the same ToBRFV isolates but has also been widely reported in isolates from other countries. As mentioned by others, the importance of cloning and characterizing novel resistance genes is paramount in developing better tomato cultivars [19]. Together, our results suggest that *Tm-1* and *RGA1* are unlikely to be solutions to the ToBRFV alone, but could be important factors in combining different mechanisms of resistance genes necessary to develop ToBRFV immune plants.

## Figures and Tables

**Table 1:** Description of tomato samples, ToBRFV detection and quantification, and *Tm-1* allele detection.

Sample ID	Greenhouse	Unknown Variety *	Labeled Resistant	*Tm-1* Allele Detection	Sampling Date	RT-PCR Detection	qPCR (ng/µL)
G1-HAR1	1	1	Yes	*Tm-1*/*tm-1*	27 September 2023	+	2.52 × 10^9^
G1-HAR2	1	2	No	*tm-1*/*tm-1*	27 September 2023	+	1.39 × 10^10^
G1-HAR3	1	2	No	*tm-1*/*tm-1*	27 September 2023	+	1.07 × 10^8^
G1-HAR4	1	2	No	*tm-1*/*tm-1*	27 September 2023	+	3.76 × 10^6^
G1-HAR5	1	2	No	*tm-1*/*tm-1*	27 September 2023	+	3.06 × 10^8^
G1-HAR6	1	2	No	*tm-1*/*tm-1*	27 September 2023	+	8.09 × 10^8^
G2-AHL1	2	3	Yes	*tm-1*/*tm-1*	27 June 2023	−	3.26 × 10^3^
G2-AHL2	2	4	No	*Tm-1*/*tm-1*	27 June 2023	−	1.16 × 10^2^
G2-TOV4	2	3	Yes	*tm-1*/*tm-1*	27 September 2023	+	1.45 × 10^8^
G2-RAV3	2	5	Yes	*tm-1*/*tm-1*	24 July 2024	+	9.64 × 10^8^
G2-GRC1	2	4	No	*tm-1*/*tm-1*	5 March 2024	+	5.36 × 10^6^
G2-TOR1	2	6	Yes	*Tm-1*/*tm-1*	20 November 2024	+	4.79 × 10^6^
G2-TOK1	2	7	Yes	*Tm-1*/*Tm-1*	20 November 2024	+	2.80 × 10^8^
G2-TOK2	2	7	Yes	*Tm-1*/*tm-1*	20 November 2024	+	1.92 × 10^7^
G3-GRT3	3	8	No	*tm-1*/*tm-1*	27 August 2024	+	2.84 × 10^9^
Qc-47044	4	9	No	Unknown	21 May 2021	+	NT
Qc-47900	4	10	No	Unknown	21 May 2021	+	NT

* Variety names were anonymized for confidentiality. NT indicates not tested.

**Table 2:** ToBRFV genomic sequencing metadata.

Sample ID	Total Reads	ToBRFV Reads	Average Depth	Average Read Length	%Coverage	Genbank Accession Number
G1-HAR1	1,793,953	6806	459.1	375.9	100	PV446608.1
G1-HAR2	740,414	4067	455.9	568.7	100	PV446609.1
G1-HAR3	239,752	4209	328.9	350.5	100	PV446610.1
G1-HAR4	114,079	4388	58.3	178.5	100	PV446611.1
G1-HAR5	101,184	3866	306.7	353.0	100	PV446612.1
G1-HAR6	66,635	4840	425.5	413.7	100	PV446613.1
G2-TOV4	659,086	5425	432.6	414.7	100	PV446618.1
G2-RAV3	67,071	3200	40.8	110.1	100	PV446614.1
G2-GRC1	197,776	4565	329.3	372.0	100	PV446606.1
G2-GRT3	193,122	5366	396.3	367.1	100	PV446607.1
G2-AHL1	530,979	3841	15.9	81.2	100	PV446604.1
G2-AHL2	393,960	4304	135.2	239.2	100	PV446605.1
G2-TOR1	554,332	89	4.1	568.8	100	PV446617.1
G2-TOK1	223,423	287	23.5	712.5	100	PV446615.1
G2-TOK2	455,294	704	41.6	611.9	100	PV446616.1
Qc-47044	85,001	1264	27.3	138.1	97.7	OQ674195.1
Qc-47900	27,868	17,307	420	155.1	100	OQ674194.1

**Table 3:** Genomic diversity of Canadian ToBRFV isolates.

ORF	n	Size (bp)	S	Non-Syn	k	h	π	FLD	FLF	TD	dN/dS	%ID
p126	20	3351	40	13	9.06	17	0.003	−1.85	−1.84	−0.94	0.20	99.5–100
p183		4848	51	14	11.8	17	0.002	−1.79	−1.61	−0.85	0.14	99.4–100
MP		801	15	8	2.90	10	0.004	−1.89	−1.95	−1.16	0.59	99.3–100
CP		480	6	0	1.35	7	0.003	−1.83	−1.85	−1.03	N/A	99.4–100
Full		6374	76	22	16.3	18	0.003	−2.02	−2.00	−1.02	0.16	99.5–100

n = number of isolates, S = number of polymorphic sites, Non-Syn = Number of non-synonymous mutations, k = average number of nucleotide differences, h = number of haplotypes, π = nucleotide diversity, FLD = Fu and Li’s D, FLF = Fu and Li’s F, TD = Tajima’s D, dN/dS = ratio of non-synonymous to synonymous substitution rates, %ID = range of nucleotide percent identities between all samples. N/A indicates not applicable, since no non-synonymous polymorphisms were identified.

**Table 4:** Non-synonymous substitutions in Canadian isolates of ToBRFV.

NT Pos.	Ref. NT	Mut. NT	AA Pos.	Ref. AA	Mut. AA	ORF	Isolates	Canadian Isolates	All Isolates
531	G	A	155	G	S	p126/p183	G2-RAV3 ^1^, G2-GRC1, G2-TOK1 ^1^, G2-TOK2 ^1^, G2-TOR1 ^1^	5	5
570	G	A	168	D	N	p126/p183	G2-RAV3 ^1^	1	1
829	A	T	254	D	V	p126/p183	G2-AHL2	1	1
1696	T	A	543	V	E	p126/p183	G2-TOV4 ^1^, G2-RAV3 ^1^, G2-AHL2, G2-GRC1, G2-TOK1 ^1^, G2-TOK2 ^1^, G2-TOR1 ^1^	7	7
1761	G	A	565	V	M	p126/p183	G1-HAR1 ^1^, G1-HAR2-6, G2-AHL1 ^1^	7	8
2338	T	C	729	L	S	p126/p183	47044	1	5
2590	A	G	841	K	R	p126/p183	G1-HAR1 ^1^, G1-HAR2-6, G2-AHL1 ^1^	7	7
**2991**	**C**	**T**	**975**	**H**	**Y**	**p126/p183**	**G2-AHL1 ^1^, G2-TOR1 ^1^**	**2**	**2**
**2993**	**C**	**A**	**975**	**H**	**Q**	**p126/p183**	**G2-TOK1 ^1^**	**1**	**1**
**2994**	**T**	**C**	**976**	**Y**	**H**	**p126/p183**	**G2-TOK2 ^1^**	**1**	**3**
**3018**	**C**	**T**	**984**	**H**	**Y**	**p126/p183**	**G1-HAR1 ^1^, G1-HAR2-6, G2-TOV4 ^1^, G2-RAV3 ^1^, G2-AHL1 ^1^, G2-AHL2, G2-GRC1, G2-TOK1 ^1^, G2-TOK2 ^1^, G2-TOR1 ^1^**	**14**	**32**
**3019**	**A**	**T**	**984**	**H**	**F**	**p126/p183**	**G2-TOV4 ^1^, G2-RAV3 ^1^, G2-AHL2, G2-GRC1, G2-TOK1 ^1^, G2-TOK2 ^1^, G2-TOR1 ^1^**	**7**	**7**
3117	G	A	1017	V	I	p126/p183	G2-GRT3 ^1^	1	2
3924	G	A	1286	A	T	p183	G2-AHL2	1	1
**5027**	**T**	**C**	**42**	**L**	**S**	**MP**	**G2-RAV3 ^1^, G2-GRC1, G2-TOK1 ^1^, G2-TOK2 ^1^, G2-TOR1 ^1^**	**5**	**5**
**5241**	**G**	**T**	**113**	**K**	**N**	**MP**	**G2-TOV4 ^1^, G2-AHL2**	**2**	**2**
**5243**	**A**	**G**	**114**	**K**	**R**	**MP**	**G2-TOV4 ^1^**	**1**	**1**
**5247**	**G**	**T**	**115**	**R**	**S**	**MP**	**G2-RAV3 ^1^, G2-GRC1, G2-TOK1 ^1^, G2-TOK2 ^1^, G2-TOR1 ^1^**	**5**	**5**
**5296**	**G**	**A**	**132**	**E**	**K**	**MP**	**G2-TOV4 ^1^, G2-RAV3 ^1^, G2-AHL2, G2-GRC1, G2-TOK1 ^1^, G2-TOK2 ^1^, G2-TOR1 ^1^**	**7**	**36**
5558	G	A	219	N	S	MP	47044	1	1
5632	G	A	244	V	I	MP	Ca1B	1	7
5680	G	A	260	V	I	MP	47900	1	119

^1^ Varieties with resistance to ToBRFV. Polymorphisms identified with major peaks in Figure 3A,B are in bold.

## Data Availability

Sequence read files are available on the sequence read archive under BioProject PRJNA1256347. Consensus sequences are available on GenBank as detailed in Table 2 and Supplemental Table S2.

## Supplementary Materials

The following supporting information can be downloaded at: https://www.mdpi.com/article/10.3390/v17050696/s1. Table S1: Primers and probe sequences. Table S2: List of all ToBRFV genomic sequences used in this analysis. Table S3: Synonymous mutations in Canadian sequences of ToBRFV.
