# Supplementary material for: Genomic Diversity of Tomato Brown Rugose Fruit Virus in Canadian Greenhouse Production Systems
Source: Viruses. 2025 May 12;17(5):696. doi: 10.3390/v17050696 (PMC12115989; doi:10.3390/v17050696)
Supplement: Supplementary file 1 [file viruses-17-00696-s001.zip › viruses-3596997-supplementary.pdf]

Supplementary Materials

Table S1. PCR and sequencing primer details.

Primer Name	Sequence
ToBRFV PCR F	AGGGCATATCCAGAATTCCA
ToBRFV PCR R	TGAGTGATCCGTACGGGATC
ToBRFV qPCR Probe[2]	56-FAM/ATACGCTGA/ZEN/AACGCCAGACGAAGT/3IABkFQ
ToB_Rp1+Adp[1]	<u>ACTTGCCTGTCGCTCTATCTTC</u>CAGGTGTTAACCCTGGTGAC
ToB_Rp2+Adp[1]	<u>ACTTGCCTGTCGCTCTATCTTC</u>CCATTGAACCCTTCATGGATG
ToB_Rp3+Adp[1]	<u>ACTTGCCTGTCGCTCTATCTTC</u>TGGACAACGCAGCGTAGTTC
ToB_Rp4+Adp[1]	<u>ACTTGCCTGTCGCTCTATCTTC</u>TCCAGCTTCTGCTTAGGTTG
ToB_Rp5+Adp[1]	<u>ACTTGCCTGTCGCTCTATCTTC</u>CTCTCCATTCTCTTATCGAC
ToB_Rp6+Adp[1]	<u>ACTTGCCTGTCGCTCTATCTTC</u>GGGGGTACCGGGGGAATTCGAA
Tm-1 FP	GAAACTATGGGCACGTTTGCAG
Tm-1 RP	CTGCACCCGCATTAGATAGA
tm-1 FP	GGAACTGTGGGCAGGTTTC
tm-1 RP	CTGCACCCGCATTAGATAGA

[1] Adaptor sequences are underlined.

[2] 3IABkFQ indicates 3′ Iowa Black FQ (IDT) quencher; 56-FAM, 5′ 6-carboxyfluorescein.

Table S2. ToBRFV Genomic sequence genbank accession information, isolate name, country and year of isolation.

Accession	Isolate Name	Country	Year	Accession	Isolate Name	Country	Year
KT383474.1	Tom1-Jo	Jordan	2015	OM515268.1	41903222	Netherlands	2021
KX619418.1	TBRFV-IL	Israel	2014	OM515269.1	38665691	Netherlands	2021
MK133093.1	TBRFV-P12-3G	Germany	2018	OM515270.1	33613331	Netherlands	2021
MK133095.1	TBRFV-P12-3H	Germany	2018	OM515271.1	41849131	Netherlands	2021
MK165457.1	Palestinian	Palestine	2018	OM515272.1	41903150	Netherlands	2021
MK319944.1	TBRFV-MX	Mexico	2018	OM718702.1	32527747	Netherlands	2021
MK648157.1	ToBRFV-CaJO	Jordan	2016	OM718703.1	41903126	Netherlands	2021
MN013187.1	F42-PAL	Palestine	2017	OM718704.1	41903310	Netherlands	2021
MN013188.1	F48-PAL	Palestine	2017	OM718705.1	41903329	Netherlands	2021
MN167466.1	ToB-SIC01/19	Italy	2018	OM718706.1	32527405	Netherlands	2021
MN182533.2	TBRFV.21930919	UK	2019	OM782671.1	ClnSin	Mexico	2019
MN549394.1	Ca1A	Canada	2019	OM892670.1	S3	USA	2020
MN549395.1	Ca1B	Canada	2019	OM892671.1	S4	USA	2020
MN549396.1	Ca2	Canada	2019	OM892672.1	S6	USA	2019
MN815773.1	ToBRFV-Gr	Greece	2019	OM892673.1	S9	USA	2019
MN882011.1	33610411	Netherlands	2019	OM892674.1	S11	USA	2021
MN882012.1	33993176	Netherlands	2019	OM892675.1	S15	Mexico	2019
MN882013.1	39976860	Netherlands	2019	OM892676.1	S17	Peru	2019
MN882014.1	36132638_A	Netherlands	2019	OM892677.1	S18	USA	2019
MN882015.1	38886177_B	Netherlands	2019	OM892678.1	S19	Peru	2019
MN882016.1	38886230_A	Netherlands	2019	OM892679.1	S20	USA	2019
MN882017.1	38886230_B	Netherlands	2019	OM892680.1	S21	USA	2018
MN882018.1	38886257_A	Netherlands	2019	OM892681.1	S23	USA	2018
MN882019.1	38887559_A	Netherlands	2019	OM892682.1	S24	USA	2018
MN882020.1	38887559_B	Netherlands	2019	OM892683.1	S25	USA	2018
MN882021.1	38887559_C	Netherlands	2019	OM892684.1	S26	Mexico	2018
MN882022.1	38890029_A	Netherlands	2019	OM892685.1	S27	Mexico	2018
MN882023.1	38890029_B	Netherlands	2019	OM892686.1	S28	Mexico	2018
MN882024.1	39055711_A	Netherlands	2019	OM892687.1	S29	Mexico	2018

MN882025.1	39055711_B	Netherlands	2019	OM892688.1	S30	Mexico	2018
MN882026.1	39058293_A	Netherlands	2019	OM892689.1	S31	Mexico	2018
MN882027.1	39058293_B	Netherlands	2019	OM892690.1	S32	Mexico	2018
MN882028.1	39070014_A	Netherlands	2019	OM892691.1	S33	Mexico	2018
MN882029.1	39070014_B	Netherlands	2019	ON528712.1	IR-Pep	Iran	2021
MN882030.1	39070022_A	Egypt	2019	OP009342.1	JoNS-Soln	Jordan	2021
MN882031.1	39070022_B	Egypt	2019	OP244618.1	wp	USA	pre-2023
MN882032.1	39070030_A	Egypt	2019	OP413740.1	PP1	Czechia	2020
MN882033.1	39070030_B	Egypt	2019	OP557566.1	ToBRFV-Ir	Iran	2021
MN882034.1	39070110_A	Netherlands	2019	OP967017.1	GBVC_ToBRFV_03	Belgium	2021
MN882035.1	39070110_B	Netherlands	2019	OP967018.1	GBVC_ToBRFV_04	Belgium	2021
MN882036.1	39070110_C	Netherlands	2019	OP967019.1	GBVC_ToBRFV_05	Belgium	2021
MN882037.1	39070153_A	Netherlands	2019	OP967020.1	GBVC_ToBRFV_06	Belgium	2021
MN882038.1	39070153_B	Netherlands	2019	OP967021.1	GBVC_ToBRFV_07	Belgium	2021
MN882039.1	39070153_C	Netherlands	2019	OP967022.1	GBVC_ToBRFV_08	Belgium	2021
MN882040.1	39070153_D	Netherlands	2019	OP967023.1	GBVC_ToBRFV_09	Belgium	2021
MN882041.1	39070153_E	Netherlands	2019	OP967024.1	GBVC_ToBRFV_10	Belgium	2021
MN882042.1	39563361_A	Netherlands	2019	OP967025.1	GBVC_ToBRFV_11	Belgium	2021
MN882043.1	39563361_B	Netherlands	2019	OP967026.1	GBVC_ToBRFV_12	Belgium	2021
MN882044.1	39563388_A	Netherlands	2019	OP967027.1	GBVC_ToBRFV_13	Belgium	2021
MN882045.1	39563388_B	Netherlands	2019	OQ091251.1	RAK_TomJB03	Lebanon	2021
MN882046.1	39941430_A	Netherlands	2019	OQ190155.1	ToBRFV-Gr2	Greece	2022
MN882047.1	39941430_B	Netherlands	2019	OQ427353.1	PEPQRO	Mexico	2022
MN882048.1	39941596_A	Netherlands	2019	OQ633211.1	402162_1	Belgium	2021
MN882049.1	39941596_B-1	Netherlands	2019	OQ633212.1	402163_1	Belgium	2021
MN882050.1	39941596_B-2	Netherlands	2019	OQ633213.1	402164_1	Belgium	2021
MN882051.1	39941617_A	Netherlands	2019	OQ633214.1	402169_1	Belgium	2021
MN882052.1	39941625_A	Netherlands	2019	OQ633215.1	402171_1	Belgium	2021
MN882053.1	39941641_A-1	Netherlands	2019	OQ633216.1	402172_1	Belgium	2021
MN882054.1	39941641_A-2	Netherlands	2019	OQ633217.1	402174_2	Belgium	2021
MN882055.1	39941641_B-1	Netherlands	2019	OQ633218.1	402175_1	Belgium	2021
MN882056.1	39941641_B-2	Netherlands	2019	OQ633219.1	402185_1	Belgium	2021

MN882057.1	39941668_A	Netherlands	2019	OQ633220.1	402275_1	Belgium	2022
MN882058.1	39941668_B	Netherlands	2019	OQ633221.1	422187_1	Germany	2021
MN882059.1	39962055_A	Netherlands	2019	OQ633222.1	422265_1	Germany	2022
MN882060.1	39962055_B	Netherlands	2019	OQ674194.1	47900	Canada	2021
MN882061.1	39962442_A	Netherlands	2019	OQ674195.1	47044	Canada	2021
MN882062.1	39962442_B	Netherlands	2019	OR437354.1	Liaoning	China	2023
MN882063.1	39963015_A	Netherlands	2019	OR451555.1	ToBRFV-XG	China	2023
MN882064.1	39963015_B	Netherlands	2019	OR500698.1	Yinchuan	China	2023
MT002973.1	CA18-01	USA	2018	OR500699.1	Zhangjiakou	China	2023
MT018320.1	ToBRFV-SD	China	2019	OR593752.1	JS-2022-ToBRFV	China	2022
MT107885.1	TBRFV-Ant-Tom	Türkiye	2019	OR760198.1	ToBRFV_G78_NRB	Netherlands	2022
MT118666.1	TBRFV-Ant-Pep	Türkiye	2019	OR760199.1	ToBRFV_G78_RB	Netherlands	2022
MW284987.1	22006291-H	France	2020	OR760200.1	472390_1	France	2023
MW314091.1	6189975_2	Netherlands	2020	OR760201.1	472392_1	France	2023
MW314092.1	32607982	Egypt	2019	OR792460.1	ToBRFV-SD	China	2020
MW314093.1	33314313_B	Egypt	2019	OR795502.1	ToBRFV-BJ	China	2020
MW314094.1	33314743	Egypt	2019	OR795503.1	ToBRFV-HB	China	2020
MW314095.1	33837271_1	Netherlands	2020	OR843983.1	MR233-17E/2703	Germany	2017
MW314096.1	33837271_2	Netherlands	2020	PP099891.1	NPPO-NL 4968182	Netherlands	2022
MW314097.1	33837296_1	Netherlands	2020	PP099892.1	NPPO-NL 36261333	Netherlands	2023
MW314098.1	33837296_2	Netherlands	2020	PP099893.1	NPPO-NL 36553581	Netherlands	2023
MW314099.1	34169391_1	Netherlands	2019	PP099894.1	NPPO-NL 36553598	Netherlands	2023
MW314100.1	34169391_2	Netherlands	2019	PP099895.1	NPPO-NL 36553651	Netherlands	2023
MW314101.1	34169402_1	Netherlands	2019	PP099896.1	NPPO-NL 36648722	Netherlands	2022
MW314102.1	34169402_2	Netherlands	2019	PP099897.1	NPPO-NL 36648757	Netherlands	2022

MW314103.1	36638057_3	Netherlands	2020	PP099898.1	NPPO-NL 38926108	Jordan	2023
MW314104.1	36689436_1	Jordan	2019	PP099899.1	NPPO-NL 39019913	Netherlands	2022
MW314105.1	36689444_1	Jordan	2020	PP099900.1	NPPO-NL 40732222	Netherlands	2023
MW314106.1	36689516_1	Netherlands	2020	PP099901.1	NPPO-NL 41008770	Spain	2022
MW314107.1	36689655	Jordan	2019	PP099902.1	NPPO-NL 41161994	Türkiye	2023
MW314108.1	36689663_1	Jordan	2019	PP099903.1	NPPO-NL 41224182	Türkiye	2021
MW314109.1	36689751_1	Jordan	2019	PP099904.1	NPPO-NL 41760970_1	Netherlands	2023
MW314110.1	36689794_1	Jordan	2019	PP099905.1	NPPO-NL 41761527	Netherlands	2023
MW314111.1	36783571_2	Peru	2020	PP099906.1	NPPO-NL 41761543	Netherlands	2023
MW314112.1	36783668_2	Netherlands	2020	PP099907.1	NPPO-NL 41761690	Netherlands	2023
MW314113.1	36783756_2	Israel	2020	PP099908.1	NPPO-NL 41761754	Netherlands	2023
MW314114.1	36783860_1	China	2020	PP099909.1	NPPO-NL 41776825	Netherlands	2022
MW314115.1	36810152_2	Israel	2020	PP099910.1	NPPO-NL 41776948_1	Peru	2022
MW314116.1	36810152_3	Israel	2020	PP099911.1	NPPO-NL 41777414	Netherlands	2023
MW314117.1	36810232_3	Netherlands	2020	PP099912.1	NPPO-NL 41777449	Netherlands	2023
MW314118.1	38589922_1	Netherlands	2020	PP099913.1	NPPO-NL 41777457	Netherlands	2023
MW314119.1	38589922_2	Netherlands	2020	PP099914.1	NPPO-NL 41777465	Netherlands	2023

MW314120.1	38589922_3	Netherlands	2020	PP099915.1	NPPO-NL 41777473	Netherlands	2023
MW314121.1	39072896_1	Netherlands	2019	PP099916.1	NPPO-NL 41780031	Netherlands	2022
MW314122.1	39072896_2	Netherlands	2019	PP099917.1	NPPO-NL 41780656	Egypt	2023
MW314123.1	39563433_3	Netherlands	2020	PP099918.1	NPPO-NL 41802579	Israel	2022
MW314124.1	39774038_1	Netherlands	2019	PP099919.1	NPPO-NL 41802683	Peru	2022
MW314125.1	39774038_2	Netherlands	2019	PP099920.1	NPPO-NL 41831994	Israel	2022
MW314126.1	39971816_1	Netherlands	2019	PP099921.1	NPPO-NL 41832073	Netherlands	2022
MW314127.1	39971816_2	Netherlands	2019	PP099922.1	NPPO-NL 41832081	Netherlands	2022
MW314128.1	39985935_4	Netherlands	2020	PP099923.1	NPPO-NL 41833738	Netherlands	2022
MW314129.1	40001980_D	Netherlands	2020	PP099924.1	NPPO-NL 41833850	Netherlands	2022
MW314130.1	40002000_A	Netherlands	2020	PP099925.1	NPPO-NL 41834028	Netherlands	2022
MW314131.1	40002166_A	Netherlands	2020	PP099926.1	NPPO-NL 41834036	Netherlands	2022
MW314132.1	40002350_A	Netherlands	2020	PP099927.1	NPPO-NL 41834079_1	Netherlands	2022
MW314133.1	40002377_1A	Netherlands	2020	PP099928.1	NPPO-NL 41834087	Netherlands	2022
MW314134.1	40002377_2B	Netherlands	2020	PP099929.1	NPPO-NL 41834140	Netherlands	2022
MW314135.1	40002377_3C	Netherlands	2020	PP099930.1	NPPO-NL 41834167	Peru	2022
MW314136.1	40002385_A	Netherlands	2020	PP099931.1	NPPO-NL 41834175	Netherlands	2022

MW314137.1	40002393_C	Netherlands	2020	PP099932.1	NPPO-NL 41834183	Peru	2022
MW349655.1	TBRFV-MX-CP	Mexico	2020	PP099933.1	NPPO-NL 41834247_1	Netherlands	2022
MZ004925.1	Y2020-3	China	2020	PP099934.1	NPPO-NL 41834255_10	Netherlands	2022
MZ202349.1	DSMZ PV-1241	Israel	pre-2021	PP099935.1	NPPO-NL 41849203	Netherlands	2021
MZ323110.1	Tom2-Jo	Jordan	2018	PP099936.1	NPPO-NL 41849246	China	2021
MZ438228.1	Tom2M-Jo	Jordan	2020	PP099937.1	NPPO-NL 41849334	China	2021
MZ945419.1	GBVC_ToBRFV_01	Belgium	2020	PP099938.1	NPPO-NL 41854424_1	Belgium	2022
MZ945420.1	GBVC_ToBRFV_02	Belgium	2020	PP099939.1	NPPO-NL 41927072_1	Peru	2022
OK339579.1	Mex2_26r	Mexico	2020	PP099940.1	NPPO-NL 41927128	Peru	2022
OK624678.1	Tom-BA21	Italy	2021	PP099941.1	NPPO-NL 41927259	Netherlands	2022
OL311702.1	DSMZ PV-1300	Cyprus	pre-2022	PP099942.1	NPPO-NL 41927291	Netherlands	2022
OM305070.1	ToBRFV-CH	Switzerland	2021	PP099943.1	NPPO-NL 41927320	Netherlands	2022
OM515230.1	2020022423	Netherlands	2020	PP099944.1	NPPO-NL 41927347	Netherlands	2022
OM515231.1	2020015323_A	UK	2020	PP099945.1	NPPO-NL 41927355	Netherlands	2022
OM515232.1	2020015323_B	UK	2020	PP099946.1	NPPO-NL 41927363	Netherlands	2022
OM515233.1	36364500_1	Peru	2020	PP099947.1	NPPO-NL 41978961	Netherlands	2022
OM515234.1	40732126_3	Israel	2020	PP099948.1	NPPO-NL 41979016	Netherlands	2021

OM515235.1	40732089_3	Peru	2020	PP099949.1	NPPO-NL 41990864	Netherlands	2022
OM515236.1	40002221_2	Netherlands	2020	PP099950.1	NPPO-NL 41992106	Netherlands	2023
OM515237.1	6166394_2	Israel	2021	PP099951.1	NPPO-NL 42039958	Netherlands	2022
OM515238.1	39986161	Netherlands	2021	PP099952.1	NPPO-NL 42284283	Netherlands	2022
OM515239.1	39986153_A-1	Netherlands	2021	PP099953.1	NPPO-NL 65661985	Netherlands	2022
OM515240.1	39986217_1	Netherlands	2021	PP099954.1	NPPO-NL 65662021_1_1	Netherlands	2022
OM515241.1	39986225_1	Netherlands	2021	PP099955.1	NPPO-NL 65662080	Peru	2022
OM515242.1	39986268	Netherlands	2021	PP099956.1	NPPO-NL 65662195_1	Netherlands	2022
OM515243.1	5678670	Netherlands	2021	PP099957.1	NPPO-NL 65662232	Netherlands	2022
OM515244.1	39986241	Netherlands	2021	PP099958.1	NPPO-NL 65662283	Netherlands	2022
OM515245.1	39986372	Netherlands	2021	PP099959.1	NPPO-NL 65662291	Netherlands	2022
OM515246.1	39986364	Netherlands	2021	PP099960.1	NPPO-NL 66046079	Netherlands	2022
OM515247.1	39986411	Netherlands	2021	PP099961.1	NPPO-NL 66046116	Netherlands	2022
OM515248.1	38950951	Netherlands	2021	PP099962.1	NPPO-NL 66046132	Netherlands	2022
OM515249.1	40002019	Netherlands	2021	PP099963.1	NPPO-NL 66046159	Netherlands	2022
OM515250.1	41106792	Israel	2021	PP099964.1	NPPO-NL 66046175	Netherlands	2022
OM515251.1	40002086	Netherlands	2021	PP099965.1	NPPO-NL 66046191_1	Netherlands	2022

OM515252.1	40002094	Netherlands	2021	PP099966.1	NPPO-NL 66047506	Netherlands	2022
OM515253.1	40001964	Netherlands	2021	PP099967.1	NPPO-NL 66047573	Netherlands	2022
OM515254.1	40001817	Netherlands	2021	PP099968.1	NPPO-NL 66048226	Netherlands	2023
OM515255.1	40001753	Netherlands	2021	PP099969.1	NPPO-NL 66048293	Netherlands	2023
OM515256.1	41106813	China	2021	PP099970.1	NPPO-NL 66048314	Netherlands	2023
OM515257.1	41108448	Israel	2021	PP099971.1	NPPO-NL 66048330	Netherlands	2023
OM515258.1	41108421	Peru	2021	PP099972.1	NPPO-NL 42457252	Morocco	2023
OM515259.1	40002289	Netherlands	2021	PP681638.1	A134T	Israel	2022
OM515260.1	40002270	Netherlands	2021	PP796738.1	ToBRFV-HB	China	2023
OM515261.1	39474756	Belgium	2021	PQ271631.1	FJ-1	Fiji	2023
OM515262.1	40002342	Netherlands	2021	PQ492144.1	ToBRFV_FJ-2	Fiji	2024
OM515263.1	40002318	Netherlands	2021	PQ492146.1	ToBRFV_GD-01	China	2024
OM515264.1	41903353	Netherlands	2021	PQ492152.1	ToBRFV_Mul-01	Pakistan	2024
OM515265.1	6165965	Belgium	2021	NC_001367.1	TMV	NCBI Reference	
OM515266.1	41106995	Israel	2021	NC_002692.1	ToMV	NCBI Reference	
OM515267.1	41903230	Netherlands	2021	NC_009041.1	RheMV	NCBI Reference	
				NC_022230.1	ToMMV	NCBI Reference	

Table S3. Synonymous mutations in Canadian isolates of ToBRFV.

NT Pos.	Ref. NT	Mut. NT	Mut. Type	AA Pos.	Ref. AA	ORF	Isolates	CAN Isolates[1]	All isolates[2]
46	T	C	N/A	N/A	N/A	5'UTR	G2-TOV4[1], G2-RAV3[1], G2-GRC1, G2-TOK1[1], G2-TOK2[1], G2-TOR1[1], 47900	7	125
50	A	T	N/A	N/A	N/A	5'UTR	G2-TOV4[1], G2-RAV3[1], G2-GRC1, G2-TOK1[1], G2-TOK2[1] G2-TOR1[1]	6	6
317	T	C	Syn	83	L	p126/p183	G2-TOV4[1], G2-RAV3[1], G2-AHL2, G2-GRC1, G2-TOK1[1], G2-TOK2[1], G2-TOR1[1]	7	8
329	A	G	Syn	87	L	p126/p183	G2-GRT3[1]	1	2
518	C	T	Syn	150	S	p126/p183	G2-GRT3[1]	1	1
551	T	C	Syn	161	N	p126/p183	G2-AHL2	1	1
608	C	T	Syn	180	H	p126/p183	G2-GRT3[1]	1	2
725	G	A	Syn	219	R	p126/p183	G1-HAR2-6, Ca2	6	17
866	C	T	Syn	266	S	p126/p183	G2-TOR1[1]	1	1
1028	G	A	Syn	320	K	p126/p183	G2-TOR1[1]	1	1
1202	C	T	Syn	378	F	p126/p183	G2-GRT3[1]	1	2
1275	T	C	Syn	403	L	p126/p183	G1-HAR1[1], G1-HAR2-6, G2-TOV4[1], G2-RAV3[1], G2-AHL1[1], G2-AHL2, G2-GRC1, G2-TOK1[1], G2-TOK2[1], G2-TOR1[1], Ca1A, Ca2	16	29
1487	T	C	Syn	473	F	p126/p183	G2-TOV4[1], G2-RAV3[1], G2-AHL2, G2-GRC1, G2-TOK1[1], G2-TOK2[1], G2-TOR1[1]	7	8
1635	T	C	Syn	523	L	p126/p183	47900	1	2
1763	G	A	Syn	565	V	p126/p183	47900	1	2
1889	T	C	Syn	607	L	p126/p183	G1-HAR1[1], G1-HAR2-6, G2-TOV4[1], G2-RAV3[1], G2-AHL1[1], G2-AHL2, G2-GRC1, G2-TOK1[1], G2-TOK2[1], Ca1A, Ca1B, Ca3, 47044	18	32
1913	A	G	Syn	615	A	p126/p183	G2-AHL2	1	1
1952	T	A	Syn	628	S	p126/p183	G2-GRT3[1]	1	1
2018	A	G	Syn	650	R	p126/p183	G2-TOV4[1], G2-RAV3[1], G2-GRC1, G2-TOK1[1], G2-TOK2[1], G2-TOR1[1]	6	6

2303	A	G	Syn	745	L	p126/p183	G1-HAR1[1], G1-HAR2-6, G2-AHL1[1]	7	7
2483	T	C	Syn	805	D	p126/p183	47900	1	3
2519	A	G	Syn	817	R	p126/p183	47900	1	115
2822	T	C	Syn	918	F	p126/p183	G2-GRT3[1]	1	1
2836	G	A	Syn	924	L	p126/p183	Ca1B	1	1
2981	C	T	Syn	971	A	p126/p183	G2-TOK1[1]	1	1
3105	T	C	Syn	1013	L	p126/p183	47900	1	119
3254	C	T	Syn	1062	I	p126/p183	G1-HAR1[1], G1-HAR2-6, G2-RAV3[1], G2-AHL1, G2-AHL2, G2-GRC1, G2-TOV4, G2-TOK1[1], G2-TOK2[1], G2-TOR1[1] Ca1A, Ca1B, Ca2, 47044	18	32
3371	T	C	Syn	1101	L	p126/p183	G1-HAR1[1], G1-HAR2-6, G2-AHL1[1]	7	7
3452	T	C	Syn	1128	S	p183	G2-TOV4[1], G2-RAV3[1], G2-AHL2, G2-GRC1, G2-TOK1[1], G2-TOK2[1], G2-TOR1[1]	7	10
3888	T	C	Syn	1274	L	p183	G1-HAR1[1], G1-HAR2-6, G2-AHL1[1], Ca1A, Ca2	9	9
4028	G	A	Syn	1320	P	p183	G2-GRT3[1]	1	3
4055	G	A	Syn	1329	S	p183	G2-RAV3[1], G2-TOK1[1]	2	9
4073	C	T	Syn	1335	I	p183	G2-GRT3[1]	1	1
4457	T	A	Syn	1463	A	p183	47900	1	44
4460	C	T	Syn	1464	S	p183	G1-HAR1[1], G1-HAR2-6, G2-AHL1[1]	7	7
4547	C	T	Syn	1493	D	p183	G2-GRT3[1]	1	1
4844	A	G	Syn	1592	V	p183	47900	1	141
4859	T	A	Syn	1597	V	p183	G1-HAR1[1], G1-HAR2-6, G2-AHL1[1]	7	7
5001	C	T	Syn	33	V	MP	G2-GRT3[1]	1	6
5127	C	T	Syn	75	G	MP	47044	1	1
5511	T	C	Syn	203	S	MP	G2-GRT3[1]	1	1
5523	A	G	Syn	207	A	MP	47044	1	2
5538	G	A	Syn	212	K	MP	G1-HAR4	1	1
5664	T	C	Syn	254	N	MP	G1-HAR1[1], G1-HAR2-6, G2-AHL1[1]	7	7
5677	T	C	Syn	255	E	MP	47900	1	1
5816	A	G	Syn	37	Q	CP	47900	1	1

5945	A	G	Syn	80	L	CP	G2-TOV4[1], G2-RAV3[1], G2-GRC1, G2-TOK1[1], G2-TOK2[1], G2-TOR1[1], Ca1B (T)	7	7
6017	A	G	Syn	104	T	CP	Ca1B	1	4
6053	T	C	Syn	116	D	CP	G1-HAR1[1], G1-HAR2-6	6	8
6098	A	G	Syn	131	V	CP	G2-GRT3[1]	1	1
6137	A	T	Syn	144	T	CP	G2-AHL2	1	2
6221	C	T	Syn	N/A	N/A	3' UTR	47900	1	4
6368	T	A	N/A	N/A	N/A	3' UTR	G1-HAR1[1], G1-HAR2-6, G2-RAV3[1], G2-AHL1, G2-AHL2, G2-GRC1, G2-TOV4, G2-TOK1[1], G2-TOK2[1], G2-TOR1[1], G3-GRT3	15	121
